# Prognostic Markers in Meningioma: Effectiveness of FISH‐Assessed *CDKN2A/B* Homozygous Deletion and Limits of Surrogate p16/MTAP Immunohistochemistry in Predicting Disease‐Specific Survival

**DOI:** 10.1111/nan.70101

**Published:** 2026-09-05

**Authors:** Alessia Andrea Ricci, Filippo Nozzoli, Alessandro Biale, Cristian Tampieri, Ludovica Verdun di Cantogno, Diego Garbossa, Mario Levis, Roberta Rudà, Camilla Bonaudo, Alessandro Della Puppa, Lorenzo Livi, Isacco Desideri, Paola Cassoni, Luca Bertero

**Affiliations:** ^1^ Pathology Unit, Department of Medical Sciences University of Turin Turin Italy; ^2^ Histopathology and Molecular Diagnostics Careggi University Hospital Florence Italy; ^3^ Pathology Unit, Department of Laboratory Medicine “Città della Salute e della Scienza” University Hospital Turin Italy; ^4^ Department of Neuroscience “Rita Levi Montalcini” University of Turin Turin Italy; ^5^ Neurosurgery Unit “Città della Salute e della Scienza” University Hospital, University of Turin Turin Italy; ^6^ Department of Oncology University of Turin Turin Italy; ^7^ Division of Neuro‐Oncology, Department of Neuroscience “Rita Levi Montalcini” University of Turin Italy; ^8^ Department of Neurosurgery Careggi University Hospital Florence Italy; ^9^ Medical and Surgical Department, Department of Neurofarba University of Florence Florence Italy; ^10^ Department of Experimental and Clinical Biomedical Sciences “M. Serio” University of Florence Florence Italy

**Keywords:** *CDKN2A/B* deletion, fluorescence in situ hybridisation, immunohistochemistry, meningioma, molecular pathology, MTAP, p16, prognosis

## Abstract

**Aims:**

Homozygous deletion (HD) of *CDKN2A/B* represents an adverse prognostic biomarker in meningiomas and a diagnostic criterion for CNS WHO grade 3 assignment. However, fluorescence in situ hybridisation (FISH) thresholds and the role of immunohistochemistry (IHC) surrogates remain uncertain. This study evaluated multiple *CDKN2A/B* FISH cutoffs and assessed the diagnostic and prognostic performance of MTAP and p16 IHC.

**Methods and Results:**

Ninety‐two meningiomas (CNS WHO grades 1–3) were analysed by FISH evaluating ≥ 10%, ≥ 20% and ≥ 30% *CDKN2A/B* HD thresholds. *CDKN2A/B* HD was identified in 18.5%, 6.5% and 4.3% of cases using the respective cutoffs and was significantly associated with shorter disease‐specific survival across all thresholds (HD ≥ 10% *p* = 0.001; HD ≥ 20% *p* = 0.0001; HD ≥ 30% *p* = 0.043). Patients with HD ≥ 20% and ≥ 30% experienced universal disease‐specific mortality within a median survival time of approximately 2 years. MTAP and p16 IHC were performed in matched areas. MTAP IHC showed high specificity (90%–92%) but limited sensitivity (30%–50%), while p16 IHC displayed variable sensitivity (41%–75%) and lower specificity (63%). Combined MTAP/p16 negativity improved sensitivity (up to 100%) but reduced specificity (57%). Neither MTAP nor p16 loss correlated significantly with survival. Spatially heterogeneous IHC patterns corresponded to regional variability in *CDKN2A/B* deletion by FISH.

**Conclusions:**

*CDKN2A/B* HD detected by FISH, particularly using thresholds equal or greater than 20%, is strongly associated with poor outcome in meningiomas. MTAP and p16 IHC, alone or combined, lack sufficient accuracy as independent surrogates but may guide tissue selection for molecular testing in heterogeneous tumours.

## Summary

- *CDKN2A/B* homozygous deletion detected by FISH is a strong adverse prognostic factor in meningiomas, particularly at thresholds ≥ 20%.
- MTAP and p16 immunohistochemistry lack sufficient accuracy to reliably substitute for direct molecular detection of *CDKN2A/B* homozygous deletion.
- Intratumoral heterogeneity may account for discordant FISH and immunohistochemical findings, supporting the use of MTAP and p16 as ancillary tools for tissue selection.

## Graphical abstract

A cohort of 92 meningiomas was reclassified according to CNS WHO 2021. Diagnostic concordance between different *CDKN2A/B* homozygous deletion cutoffs assessed by FISH and MTAP/p16 immunohistochemistry was evaluated, with the aim to improve meningioma diagnostic workflow and prognostic estimation.

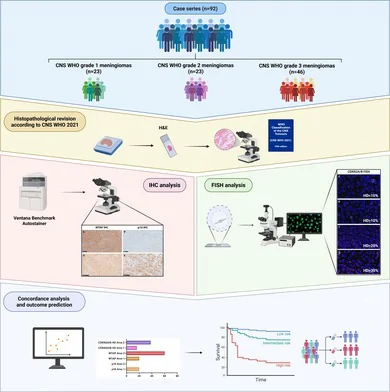

**Abbreviations**
AWD: alive with disease
CNV: copy‐number variation
DOC: dead of other causes
DOD: dead of disease
DSS: disease‐specific survival
FISH: fluorescence in situ hybridisation
H&E: haematoxylin and eosin
HD: homozygous deletion
HetD: heterozygous deletion
IHC: immunohistochemistry
NED: no evidence of disease
SD: standard deviation

## 1 Introduction

Meningiomas represent the most common primary intracranial tumours (42.6% of all CNS tumours and 57.4% of nonmalignant lesions) [1]. Their clinical behaviour is highly heterogeneous, ranging from indolent to aggressive disease [2]. According to the fifth edition of the WHO Classification of the CNS Tumours (CNS WHO 2021), meningiomas are divided into several histological subtypes and graded into three malignancy grades that correlate with recurrence risk and survival [2]. However, histology‐based grading is affected by considerable inter‐observer variability, prompting increasing reliance on molecular pathology to refine prognostic stratification and guide therapeutic decisions [3].

Among molecular biomarkers, homozygous deletion (HD) of the *CDKN2A/B* locus on chromosome 9p21 and *TERT* promoter mutations have emerged as strong predictors of poor outcome [4, 5, 6, 7] and are now independent diagnostic criteria for CNS WHO grade 3 meningiomas [2]. Additionally, the cIMPACT‐NOW consortium proposed further molecular criteria to refine CNS WHO grade 2 classification, including 1p deletions in the context of monosomy 22 or *NF2* alterations [3]. Consequently, molecular profiling has become pivotal for meningioma classification and management.

*CDKN2A/B* deletions lead to loss of the tumour suppressors *p16* ^*INK4a*^ and *p14* ^*ARF*^, promoting dysregulated cell cycle progression [8]. Notably, a recent multicentre study further validated the prognostic value of *CDKN2A/B* HD in multivariable analysis, whereas *TERT* promoter status did not retain independent significance [9]. Accurate detection of *CDKN2A/B* HD is therefore increasingly relevant in routine diagnostics. Although DNA methylation profiling enables simultaneous assessment of methylation classes and copy‐number variation (CNV), its widespread use is limited by cost and availability. Thus, fluorescence in situ hybridisation (FISH) remains a reference method for CNV detection, but is time‐consuming, costly and not universally accessible. These limitations have driven interest in immunohistochemical (IHC) surrogate markers, especially loss of key tumour suppressor p16 [10]. However, p16 is frequently absent in low‐grade meningiomas, and its diagnostic performance varies across studies [11, 12, 13, 14, 15]. Attention has therefore shifted to MTAP, encoded by a gene adjacent to *CDKN2A/B* and commonly co‐deleted. Several studies have demonstrated strong concordance between MTAP IHC loss and *CDKN2A/B* HD in meningiomas [12, 13, 14, 16], although most were limited by small cohort sizes. To date, the comparative diagnostic performance of MTAP and p16 IHC has not been clearly established.

In this study, we aimed to define the optimal FISH cutoff for *CDKN2A/B* homozygous deletion (i.e., the minimum proportion of tumour cells showing *CDKN2A/B* HD used to classify a case as homozygously deleted) and to evaluate and compare MTAP and p16 IHC as surrogate markers across CNS WHO grade 1–3 meningiomas, with the goal of improving diagnostic workflows.

## 2 Materials and Methods

### 2.1 Case Selection

Initially, all samples from CNS WHO grade 1, 2 and 3 meningiomas resected between 2006 and 2021 with both adequate histological material and matched clinical data were retrieved from the pathology files of the ‘Città della Salute e della Scienza’ Hospital (Turin, Italy) and the Careggi University Hospital (Florence, Italy). More recent samples were not included to ensure adequate follow‐up. Representative formalin‐fixed and paraffin‐embedded (FFPE) tissue samples were selected for each case included in the study and submitted to histopathological review according to CNS WHO 2021 [2]. A series representative in terms of WHO grade was then randomly included in the study and submitted to further analysis, prioritising the collection of CNS WHO grade 3 meningiomas, given the higher frequency of *CDKN2A/B* HD in this group, yielding a 1:1:2 ratio according to WHO grade 1, 2 and 3 neoplasms, respectively. Cases were graded according to CNS WHO 2021 criteria; for grading purposes, a FISH cutoff value of homozygous deleted cells ≥ 30% was used, in accordance with previous studies, including studies of other CNS tumours (Table S1) [17]. Clinical data such as age at diagnosis, sex, tumour location and follow‐up data were retrieved from patients' clinical reports in a dedicated database.

### 2.2 CDKN2A/B Locus FISH Analysis

Based on H&E staining, a tumour area was selected for FISH analysis in accordance with routine practice, focusing on areas suggestive of greater aggressiveness (e.g., higher cellularity, higher mitotic activity and presence of necrosis) and with adequate quality (e.g., avoiding tissue artefacts). Sections 4 μm thick were cut and FISH was performed using the Zytolight SPEC CDKN2A/CEN 9 Dual Colour probe (Zytovision, Bremerhaven, Germany) according to the manufacturer's instructions. Hybridisation was performed using a HyBrite (Abbott Molecular Inc., IL, USA) and slides were analysed using the fluorescence microscopes GSL120 Leica (Leica Biosystems Richmond Inc., IL, USA) and Axio Imager Z1 (ZEISS, Oberkochen, Germany). At least 10 representative areas per case were selected and automatically acquired at ×63 magnification, counting 100 nonoverlapping nuclei to determine the following categories in line with previous studies [17, 18]: normal (two *CDKN2A/B* and two centromeric signals); heterozygous deletion (HetD) (loss of one copy of *CDKN2A/B* and two centromeric signals); homozygous deletion (HD) (loss of two copies of *CDKN2A/B* and one or two centromeric signals; when only one centromeric signal was present, attention was paid to the overall nuclear morphology and image quality to minimise the risk of potential artefacts).

### 2.3 Immunohistochemistry

Sections 3 μm thick were cut from the same FFPE tissue block used for *CDKN2A/B* FISH analysis for each case. IHC was performed on the automated platform Ventana BenchMark AutoStainer (Ventana Medical Systems, AZ, USA) according to the manufacturer's instructions with the following primary antibodies: MTAP (monoclonal antibody, clone 2G4, diluted 1:150; Abnova, Taipei City, Taiwan) and CINtec p16 Histology (monoclonal antibody, prediluted; Ventana Medical Systems, AZ, USA). MTAP retention was defined as cytoplasmic expression with or without nuclear staining in tumour cells, and MTAP loss as the absence of cytoplasmic expression in tumour cells with endothelial cells serving as a positive internal control. p16 antibody staining was considered positive for nuclear and/or cytoplasmic localisation. A cutoff of < 10% positive cells was used to define samples as negative by IHC. When used in combination, staining with < 10% positive cells for at least one marker was considered sufficient to classify the sample as negative. Of note, IHC staining was evaluated in the same areas analysed by FISH and IHC slides were compared with the H&E section cut at the time of FISH analysis to exclude significant changes due to sample consumption which could contribute to discrepant results.

### 2.4 Statistical Analysis

All analyses were performed using Stata/MP 18.0 statistical software (STATA, College Station, TX, USA), SPSS Inc. (IBM, Chicago, IL, USA) and Prism GraphPad 9.0 (GraphPad Software, San Diego, CA, USA). Continuous variables were summarised as the mean and standard deviation (SD), whereas categorical variables were reported as frequencies. IHC and FISH results were compared using the chi‐square test and Cohen's kappa coefficient for categorical variables. Disease‐specific survival (DSS) was calculated from the date of diagnosis to the date of death, if available, or censored at date of last follow‐up. Median follow‐up was estimated according to the reverse Kaplan–Meier method. Patients' outcomes were categorised as dead of disease (DOD), dead of other causes (DOC), alive with disease (AWD) or no evidence of disease (NED), and only deaths attributable to the disease (DOD) were considered as events for DSS analysis. Survival curves between different groups were plotted using the Kaplan–Meier method, and the statistical comparisons were performed using the log‐rank test. Cox regression analyses were conducted on DSS to calculate HRs and 95% CIs for the different study groups. DSS was then assessed using the Aalen–Johansen method to appropriately account for competing risks—such as death without recurrence—providing an unbiased estimate of the cumulative incidence of disease‐specific death, as previously published [18]. Cumulative incidence functions (CIFs) were computed according to *CDKN2A/B* status (homozygous deletion [HD] vs. nonhomozygous deletion [non‐HD]) and further stratified by age (< 55, 55–69 and ≥ 70 years). Pointwise 95% confidence intervals (95% CIs) were derived via Greenwood's variance. Log‐rank tests were used to compare groups within age strata, and a Mantel–Haenszel age‐stratified log‐rank test provided the overall comparison of HD versus non‐HD tumours. Median follow‐up was estimated using the reverse Kaplan–Meier method.

## 3 Results

### 3.1 Patients' Baseline Characteristics

Ninety‐two patients diagnosed with CNS WHO grade 1, 2 and 3 meningiomas were included in this study. Fifty of 92 patients (54.3%) were female and 42 (45.7%) were male; the median age was 67 years (range 19–85), and the median follow‐up was 69.3 months (range 0.1–214.2). The cohort was stratified as follows: 23/92 (25%) CNS WHO grade 1 meningiomas, 23/92 (25%) CNS WHO grade 2 meningiomas and 46/92 (50%) CNS WHO grade 3 meningiomas.

### 3.2 CDKN2A/B HD FISH Analysis

As reported in the Methods section, even though a *CDKN2A/B* homozygous deletion FISH cutoff of ≥ 30% was used for WHO grading purposes, different cutoff values of ≥ 10%, ≥ 20% and ≥ 30% deleted cells were assessed by FISH as potential thresholds to define a sample as homozygous deleted. According to the 10% cutoff, 17/92 (18.5%) cases were HD for *CDKN2A/B*, specifically, 1/23 (4.3%) CNS WHO grade 1 meningioma, 5/23 (21.7%) CNS WHO grade 2 meningiomas and 11/46 (23.9%) CNS WHO grade 3 meningiomas (*p* = 0.126). According to the 20% cutoff, the total number of *CDKN2A/B* HD meningiomas was 6/92 (6.5%), of which 5/46 (10.8%) were CNS WHO grade 3 meningiomas and 1/23 (4.3%) was a CNS WHO grade 2 meningioma (*p* = 0.304). According to the 30% cutoff, only 4/92 (4.3%) cases were HD for *CDKN2A/B* and all were CNS WHO grade 3 meningiomas (4/46; 8.7%) (*p =* 0.175).

### 3.3 Disease‐Specific Survival Analysis According to CDKN2A/B Status

**FIGURE 1:**
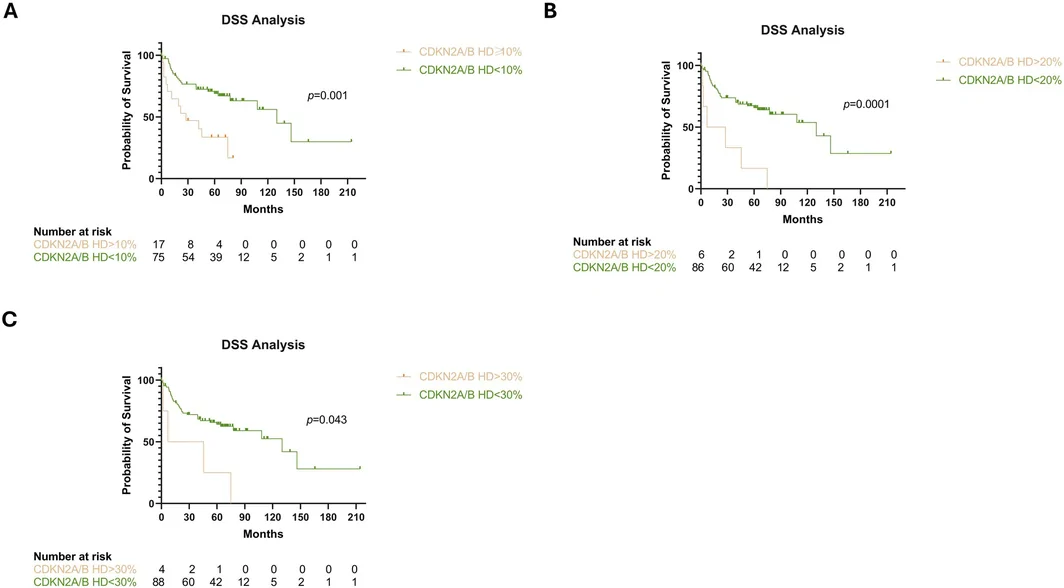
DSS KM analysis according to *CDKN2A/B* HD ≥ 10% (A), ≥ 20% (B) and ≥ 30% (C) cutoff values.

The median DSS among deaths was 18.4 months (range 0.9–146.2), with 53/92 (57.6%) patients alive at the end of follow‐up (August 2025) and 39/92 (42.4%) patients who died of disease (DOD). No deaths from other causes (DOCs) were considered in the present analysis, as only disease‐related deaths (DODs) were counted as events. According to *CDKN2A/B* status, all patients with HD ≥ 20% and HD ≥ 30% died of disease within a median survival time ranging from 17.4 to 26.3 months, while all surviving patients were classified as non‐HD for these cutoffs (*p* = 0.003; *p* = 0.029). Five out of 17 (29.4%) patients with *CDKN2A/B* HD ≥ 10% were still alive at the end of the analysis (*p* = 0.009). Median DSS of *CDKN2A/B* HD DOD patients versus non‐HD was 15.4 months (range 1.9–74.8) versus 18.4 months (range 0.9–146.2) for the ≥ 1 0% cutoff (*p* = 0.31), 17.4 months (range 1.9–74.8) versus 18.4 months (range 0.9–146.2) for the ≥ 20% cutoff (*p* = 0.61) and 26.3 months (range 1.9–74.8) versus 18.4 (range 0.9–146.2) for the ≥ 30% cutoff (*p* = 0.68). Nevertheless, a significant association with shorter disease‐specific survival in the log‐rank test emerged for all tested cutoffs (HD ≥ 10% *p* = 0.001; HD ≥ 20% *p* = 0.0001; HD ≥ 30% *p* = 0.043) (Figure 1 and Figure S1).

At a median follow‐up of 69.3 months, DSS differed across *CDKN2A/B* status and age strata. According to the Aalen–Johansen estimator, the estimated cumulative incidence of disease‐specific death (DSD) at 144 months was 100.0% (95% CI, 100.0–100.0) among patients harbouring a *CDKN2A/B* HD ≥ 30% aged 55–69 years (*n = 3; 3 events*) and 100.0% (95% CI, 100.0–100.0) among those aged ≥ 70 years (*n = 1; 1 event*). No HD cases were present in the < 55‐year group, precluding estimation for that stratum. In contrast, non‐HD meningiomas exhibited substantially lower cumulative incidence rates across younger age groups.

The estimated probability of DSD at 144 months was 14.0% (95% CI, 5.2–37.6) among patients aged < 55 years (*n = 20; 4 events*), 43.8% (95% CI, 26.1–66.5) among those aged 55–69 years (*n = 30; 13 events*) and 72.0% (95% CI, 54.6–87.4) among patients aged ≥ 70 years (*n = 38; 18 events*).

**FIGURE 2:**
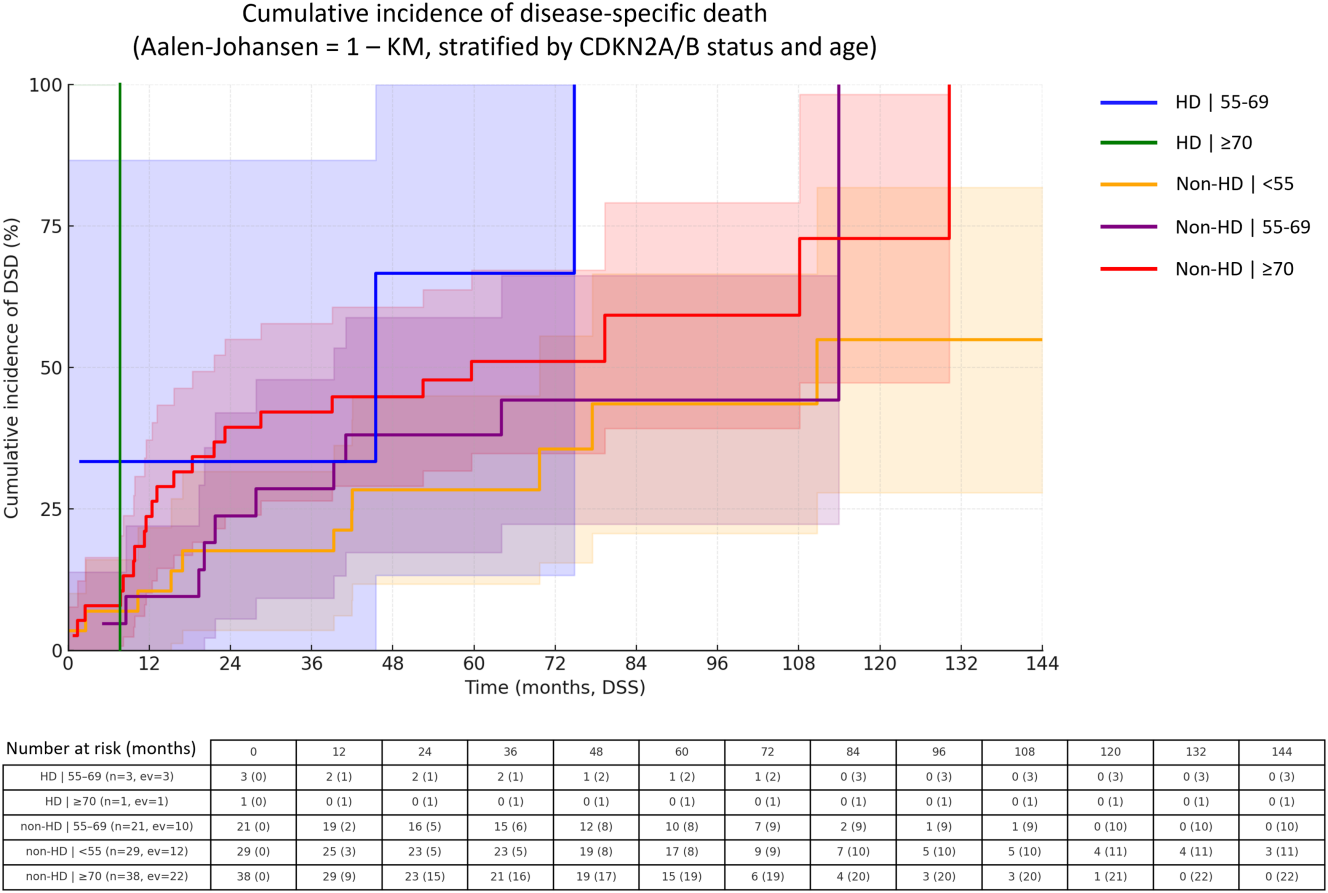
Cumulative incidence functions for disease‐specific death (Aalen–Johansen method with pointwise 95% confidence intervals) stratified by *CDKN2A/B* status and age group. Corresponding risk tables are shown below each panel.

When comparing HD and non‐HD tumours within age strata, a significant difference in DSD rates was observed among older patients (≥ 70 years; χ^2^(1) = 7.68, *p* = 0.006), whereas no significant difference emerged in the 55–69‐year group (χ^2^(1) = 0.23, *p* = 0.63). The comparison could not be performed for patients younger than 55 years due to the absence of HD cases. In the age‐stratified log‐rank test pooling all age groups, the overall difference between HD and non‐HD meningiomas did not reach statistical significance (χ^2^(1) = 1.49, *p* = 0.22), although the results suggested a trend toward worse DSS among older patients with *CDKN2A/B* homozygous deletions (Figure 2).

Additionally, we investigated the effect of age, grade and *CDKN2A/B* HD upon DSS using Cox regression analysis. Age (HR = 2.6; 95% CI, 1.13–6.03; *p* = 0.025), higher histological grade (HR = 17.1; 95% CI, 5.24–55.8; *p* < 0.001), CDKN2A/B HD ≥ 10% (HR = 3.05; 95% CI, 1.52–6.14; *p* = 0.002), HD ≥ 20% (HR = 4.73; 95% CI, 1.96–11.45; *p* = 0.001) and HD ≥ 30% (HR = 4.06; 95% CI, 1.43–11.52; *p* = 0.009) showed a negative impact on survival in univariate analysis (Table 1).

**TABLE 1:** Univariate Cox proportional hazards analysis according to DSS.

		HR	CI	p
Age	Linear	2.7	1.16–6.23	0.021
Sex	M vs. F	1.14	0.61–2.14	0.687
CDKN2A/B HD	≥ 10%	2.8	1.41–5.62	0.003
	≥ 20%	3.73	1.54–9.05	0.004
	≥ 30%	3.01	1.05–8.67	0.041
Grade	1/2 vs. 3	16.3	4.99–53.1	< 0.001
MTAP	< 10%	1.51	0.63–3.64	0.355
p16	< 10%	0.81	0.42–1.58	0.541
MTAP/p16	< 10%	0.84	0.44–1.59	0.591

*Note:* Significance: *p* < 0.05.

### 3.4 Immunohistochemistry Evaluation and Correlation With FISH Analysis

**FIGURE 3:**
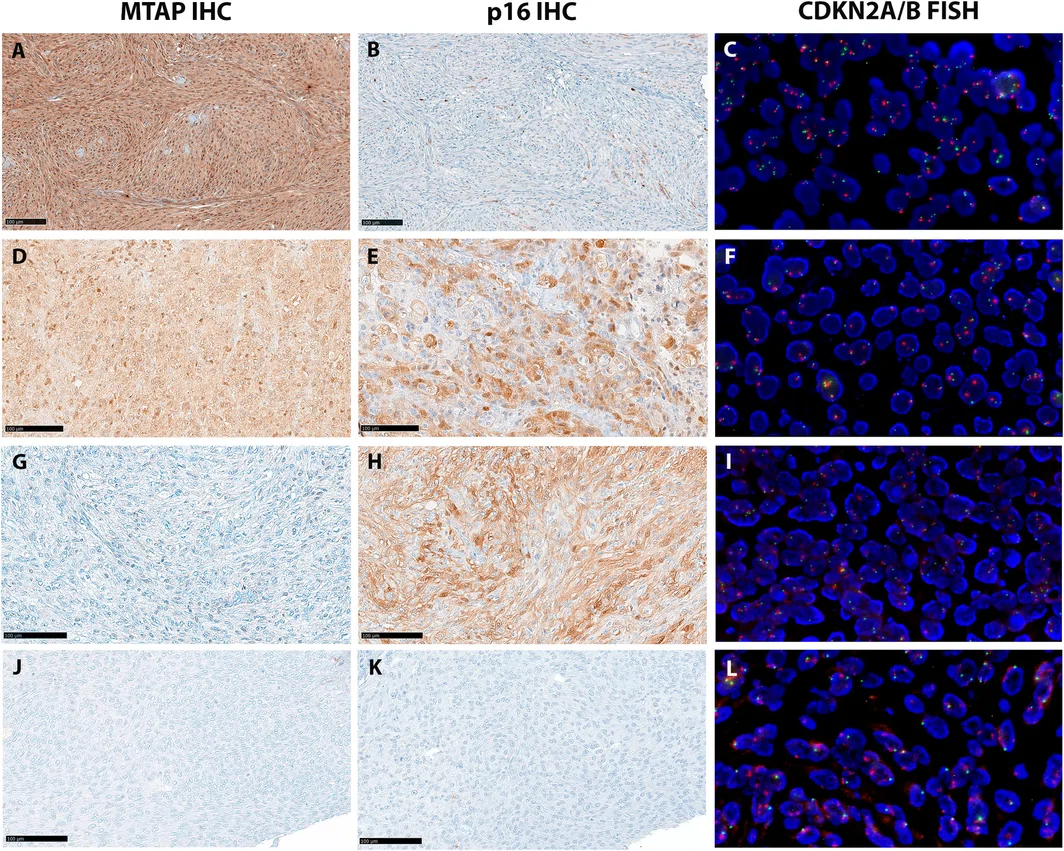
Examples of the evaluated *CDKN2A/B* HD FISH thresholds and the corresponding MTAP and p16 staining patterns. (C) *CDKN2A/B* non‐HD with concordant positive MTAP IHC (A) and discordant p16 (B). (F) CDKN2A/B HD ≥ 10% with concordant positive MTAP (D) and p16 (E) staining. (I) *CDKN2A/B* HD ≥ 20% with concordant negative MTAP staining (G) and discordant p16 (H). (L) *CDKN2A/B* HD ≥ 30% with concordant negative MTAP (J) and p16 (K) staining.

MTAP and p16 IHC were evaluated by two independent pathologists in the same slide selected for FISH analysis according to the criteria reported in the Methods section. In case of disagreement, a consensus was reached by joint review and discussion. While p16 and MTAP immunohistochemistry were performed on whole tissue sections, their assessment was limited to the same tumour area selected for FISH analysis. Only the p16/MTAP staining pattern within this corresponding region was used for correlation with FISH, ensuring that the molecular and immunohistochemical evaluations were spatially aligned and referred to the same portion of the tumour. Both markers' sensitivity and specificity were assessed in relation to *CDKN2A/B* status, either alone or in combination (Table 2). Overall, 11/92 (11.9%) and 35/92 (38%) cases showed negative MTAP and p16 staining, respectively. Of the 17 cases harbouring *CDKN2A/B* HD ≥ 10%, 5 (29.4%) were also MTAP negative (*p* = 0.014; sensitivity 29.41%, specificity 92%) and 7 (41.2%) p16 negative (*p* = 0.768; sensitivity 41.2%, specificity 62.7%). For *CDKN2A/B* HD ≥ 20%, 3/6 (50%) cases showed negative MTAP (*p =* 0.021; sensitivity 50%, specificity 90.7%), and p16 (*p =* 0.670; sensitivity 50%, specificity 62.8%) staining, whereas among the 4 *CDKN2A/B* HD ≥ 30% cases, 2/4 (50%) were MTAP negative (*p =* 0.069; sensitivity 50%, specificity 89.8%) and 3/4 (75%) p16 negative (*p =* 0.152; sensitivity 75%, specificity 63.6%). Figure 3 illustrates examples of the evaluated *CDKN2A/B* HD cutoffs with the corresponding MTAP and p16 staining patterns. Subsequently, the combined performance of the two markers was evaluated in relation to *CDKN2A/B* status to determine whether their joint use could improve their accuracy. Results differed slightly from those obtained with individual marker use: the HD cases also negative for IHC were 10/17 (58.8%) for the ≥ 10% cutoff (*p* = 0.227; sensitivity 58.8%, specificity 57.3%), 5/6 (83.3%) for the ≥ 20% cutoff (*p* = 0.089; sensitivity 83.3%, specificity 57%) and 4/4 for the ≥ 30% cutoff (*p* = 0.040; sensitivity 100%, specificity 56.8%). However, concordance between MTAP and p16 staining was observed in only 54/92 (58.7%) cases (Cohen's kappa coefficient = −0.010), indicating no significant agreement beyond chance.

**TABLE 2:** MTAP, p16 and MTAP/p16 performance according to different *CDKN2A/B* HD cutoffs.

	*CDKN2A/B* HD cutoff	Sensitivity	Specificity	p
MTAP	≥ 10%	29.41%	92%	0.014
	≥ 20%	50%	90.7%	0.003
	≥ 30%	50%	89.8%	0.016
p16	≥ 10%	41.18%	62.7%	0.768
	≥ 20%	50%	62.8%	0.533
	≥ 30%	75%	63.6%	0.120
MTAP/p16	≥ 10%	58.8%	57.3%	0.227
	≥ 20%	83.3%	57%	0.055
	≥ 30%	100%	56.8%	0.026

*Note:* Significance: *p* < 0.05.

### 3.5 Disease‐Specific Survival Analysis According to MTAP and p16 Status

**FIGURE 4:**
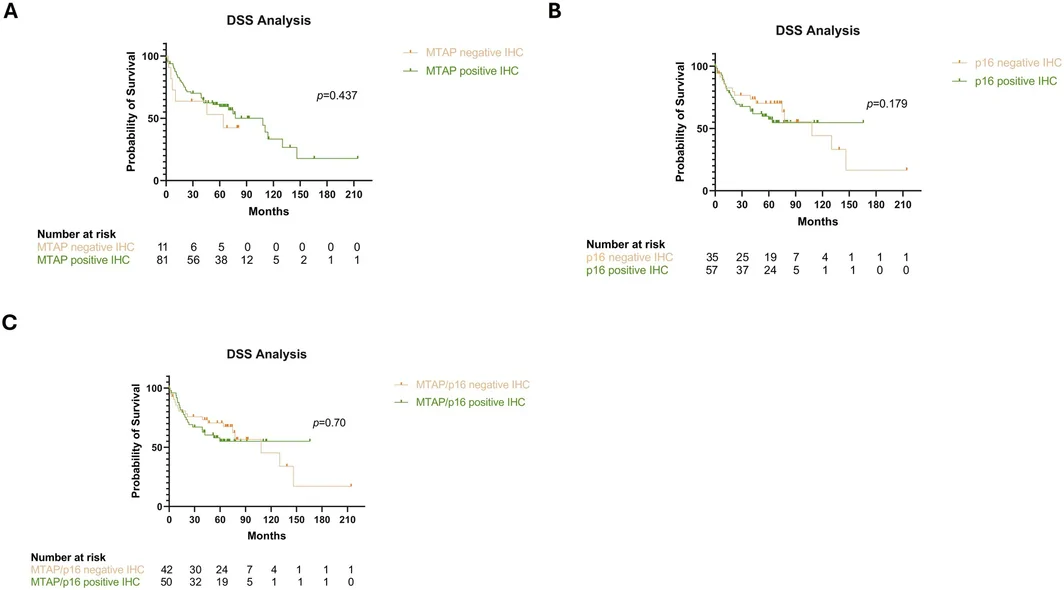
DSS KM analysis according to MTAP (A), p16 (B), and MTAP/p16 (C) IHC.

Of the 39 patients who had died of disease at the end of the analysis, 6 (15.4%) were MTAP negative (*p* = 0.385) and 15 (38.5%) p16 negative (*p* = 0.944). When combined, 18/39 cases had negative IHC (46.2%) (*p* = 0.934). Median DSS in positive versus negative IHC cases was 21.6 months (range 0.9–146.2) versus 8.7 months (range 2.6–64) for MTAP (*p* = 0.97); 16.1 months (range 1.4–64) versus 21.6 months (range 0.9–146.2) for p16 (*p* = 0.17); 16.9 months (range 1.4–59.7) versus 20.5 (range 0.9–146.2) considering MTAP and/or p16 (*p* = 0.97). No immunohistochemical surrogate marker, assessed either individually or in combination, was found to be significantly associated with disease‐specific survival in the log‐rank test (MTAP *p* = 0.437; p16 *p* = 0.179; MTAP/p16 *p* = 0.70) (Figure 4), nor in univariate Cox proportional hazards analysis (Table 1).

### 3.6 FISH and IHC Spatial Heterogeneity Assessment

In a subset of five cases showing marked heterogeneity in IHC staining, FISH analysis was repeated on two distinct regions of the same tissue block (Figure S2). These were selected based on either MTAP or p16 IHC when the difference in staining intensity between areas was > 20%. Three cases were selected according to p16 staining, one according to MTAP staining and the final case showed heterogeneity for both markers. In two cases (TO3 and TO15), *CDKN2A/B* HD status differed markedly between the two areas, showing good concordance with p16 but not with MTAP IHC.

## 4 Discussion

In this study, the prognostic impact of different thresholds for *CDKN2A/B* HD was assessed in a large cohort of meningiomas. *CDKN2A/B* loss emerged as a strong adverse prognostic factor for DSS, particularly when a deletion threshold of ≥ 20% was applied, confirming its role as a robust and independent marker of aggressive behaviour. In contrast, IHC surrogate markers MTAP and p16 showed variable concordance with *CDKN2A/B* status and failed to reliably predict survival, underscoring the limitations of IHC‐based approaches in this context.

Accurate identification of meningiomas harbouring unfavourable molecular profiles is increasingly relevant in light of the updated WHO classification. The negative prognostic impact of *CDKN2A/B* HD has been consistently demonstrated in multiple large cohorts, although most studies relied on genome‐wide approaches such as methylation profiling or next‐generation sequencing (NGS). In contrast, data derived from FISH, including validated cutoff values, remain limited and heterogeneous [13, 14, 19]. To address this gap, we performed a stepwise evaluation of three incremental FISH thresholds (≥ 10%, ≥ 20% and ≥ 30%) to define biologically and clinically meaningful *CDKN2A/B* HD.

All tested thresholds were significantly associated with adverse outcome, with prognostic relevance maintained even at the highest cutoff despite the limited number of cases. Notably, all patients harbouring *CDKN2A/B* HD ≥ 20% died within approximately 2 years of diagnosis, further supporting the clinical impact of this alteration [5, 6]. These findings are consistent with those of Li et al., who identified 23% as the optimal cutoff to maximise concordance between FISH and NGS results [20]. Moreover, cumulative incidence analysis revealed a significantly higher disease‐specific mortality in patients aged ≥ 70 years with HD ≥ 30% compared to non‐HD cases, suggesting a possible age‐dependent effect of *CDKN2A/B* loss on tumour aggressiveness. Based on these observations and to minimise technical noise related to truncated nuclei or signal loss, we propose adopting a more stringent threshold (e.g., ≥ 30%), as already recommended for other CNS tumours [17].

Given the technical and interpretative challenges associated with FISH, reliable IHC surrogates for *CDKN2A/B* HD would represent a major practical advantage in routine diagnostics. Both p16 and MTAP IHC have been extensively investigated as surrogate markers, but published results remain inconsistent [11, 12, 13, 15, 16, 19, 21, 22]. While p16 loss has been proposed as a common finding in CNS WHO grade 1 and 2 meningiomas [15], some studies reported high sensitivity and specificity in malignant tumours, although the number of high‐grade cases harbouring *CDKN2A/B* HD was often small [11]. Instead, MTAP has been suggested to outperform p16 in terms of specificity and may potentially serve as a standalone surrogate [13, 14], although other studies cautioned against relying on MTAP alone [19].

In our cohort, both markers showed variable diagnostic performance depending on the applied deletion threshold. MTAP IHC demonstrated high specificity (89.8%–92%) but modest sensitivity (29.4%–50%), whereas p16 showed highly variable sensitivity (41.2%–75%) and moderate specificity (62.7%–63.6%). These findings align with prior evidence that p16 expression can be lost through mechanisms unrelated to gene deletion [12, 13], particularly in lower‐grade tumours where baseline expression is often minimal [19]. Conversely, MTAP loss appears more specific for true deletions but may miss partial or heterogeneous alterations that spare the MTAP locus [13, 16]. Combining MTAP and p16 did not improve overall accuracy, yielding only marginal gains in sensitivity at the highest deletion cutoff (58.8%–100%) at the expense of a substantial loss of specificity (56.0%–57.3%).

Intratumoral heterogeneity further complicates the use of IHC surrogates. Previous reports described subclonal *CDKN2A/B* and *MTAP* deletions associated with regionally distinct staining patterns [19], consistent with known spatial genetic heterogeneity in meningiomas [23, 24, 25]. Our own validation of discrepant regions confirmed variable correlations between IHC and FISH, with p16 showing partial concordance in a few cases. Despite a higher proportion of deceased patients showing MTAP loss, neither MTAP nor p16 IHC was significantly associated with disease‐specific survival. This suggests that IHC markers do not adequately capture the biological aggressiveness conferred by *CDKN2A/B* HD, limiting their prognostic utility. These discrepancies align with prior observations that concordance between IHC, FISH and molecular methods is highly dependent on threshold selection and tumour heterogeneity [20]. Even though combined MTAP and p16 staining did not enhance diagnostic performance overall, their joint use may retain practical value in guiding tissue selection for molecular testing in heterogeneous tumours [12, 13].

Although the number of *CDKN2A/B* HD‐positive cases appears limited in our series, its prevalence at the ≥ 30% threshold is consistent with published rates (< 5%) [3, 7]. Importantly, our cohort represents one of the largest series to date enriched for CNS WHO grade 3 meningiomas, providing a more robust basis for analysis compared with prior studies analysing 3–15 CNS WHO grade 3 meningiomas [11, 12, 13, 14, 16]. A further limitation could be the use of FISH as a reference assay, as it represents a single‐cell–level quantitative technique; however, recent comparative studies demonstrated high concordance between FISH and bulk methods such as NGS for *CDKN2A/B* HD detection, supporting its reliability despite inherent technical constraints [20, 22].

In conclusion, *CDKN2A/B* HD is confirmed as a clinically meaningful adverse prognostic factor in meningiomas, particularly at thresholds ≥ 20%. In contrast, MTAP and p16 IHC show inconsistent correlation with molecular status and no independent prognostic value. While MTAP may retain limited utility due to higher specificity, neither marker can replace direct molecular testing, though both may serve as ancillary tools for tissue selection in the context of intratumoral heterogeneity.

## Author Contributions

P.C. and L.B. designed the study. A.A.R. and F.N. conducted data analysis and wrote the manuscript. A.A.R. performed H&E and IHC staining. F.N. and A.B. were responsible for histological revision and immunohistochemistry evaluation. C.T. and L.V.C. carried out FISH analysis. D.G., M.L., R.R., C.B., A.D.P., L.L. and I.D. provided clinical data for the study. All authors reviewed and approved the final manuscript.

## Funding

This study was supported by research grants from Fondazione Ricerca Molinette ETS, Turin, Italy, Fondazione CRT (Grant reference number: 113265/2025.0323), Turin, Italy, and Rete Oncologica Piemonte e Valle d'Aosta, Turin, Italy to L.B.

## Ethics Statement

The study was conducted in accordance with the ethical standards of the University of Turin IRB and with the Code of Ethics of the World Medical Association (Declaration of Helsinki and following amendments). The Florence cohort was included in this study according to the Local Ethics Committee of Tuscany (Protocol Number: 27080_BIO).

## Consent

Informed consent to the surgical procedure and data collection was obtained from all subjects involved in the study, while dedicated written informed consent was unnecessary because of the retrospective nature of the study.

## Conflicts of Interest

The authors declare no conflicts of interest.

## Supporting information


**Figure S1:** DSS KM cumulative analysis according to all *CDKN2A/B* HD tested cutoff values.

**Figure S2:** FISH and IHC spatial heterogeneity assessment and quantification in multiple areas of samples TO3, TO15, TO59, TO60 and TO74.

**Table S1:** Features of CNS WHO grade 3 meningiomas according to CNS WHO 2021. Seventeen CNS WHO grade 3 meningiomas (34%) were classified based solely on mitotic count, while three cases (6%) exhibited overt anaplasia as the only criterion. Three additional cases were reclassified based on molecular features: one due to *CDKN2A/B* HD ≥ 30%, one due to the presence of a *TERT* promoter pathogenic mutation and one that harboured both alterations.

## Data Availability

The collected/analysed data are not publicly available to protect patients' privacy and comply with ethical requirements. Aggregated data supporting the study findings are available from the corresponding author upon a reasonable request.
