# Supplementary material for: Prognostic Markers in Meningioma: Effectiveness of FISH‐Assessed CDKN2A/B Homozygous Deletion and Limits of Surrogate p16/MTAP Immunohistochemistry in Predicting Disease‐Specific Survival
Source: Neuropathol Appl Neurobiol. 2026 Sep 5;52(5):e70101. doi: 10.1111/nan.70101 (PMC13545683; doi:10.1111/nan.70101)
Supplement: Supplementary file 1 [file NAN-52-e70101-s001.docx]

*Table S1.* Features of CNS WHO grade 3 meningiomas according to CNS WHO 2021.

Seventeen CNS WHO grade 3 meningiomas (34%) were classified based solely on mitotic count, while 3 cases (6%) exhibited overt anaplasia as the only criterion. Three additional cases were reclassified based on molecular features: one due to *CDKN2A/B* HD≥30%, one due to the presence of a *TERT* promoter pathogenic mutation, and one that harboured both alterations.

| ID | Mitosis/10 HPF of 0.16 mm2 | Overt anaplasia | *CDKN2A/B* HD≥30% | *TERT* promoter mutation |
| --- | --- | --- | --- | --- |
| TO1 | 11 | - | Yes | - |
| TO2 | 11 | - | - | - |
| TO3 | 21 | - | - | Yes |
| TO4 | 20 | - | - | Yes |
| TO5 | 22 | - | - | - |
| TO6 | 30 | - | - | - |
| TO7 | 22 | - | - | - |
| TO8 | 22 | Yes | - | - |
| TO9 | 28 | - | - | - |
| TO10 | 22 | - | - | - |
| TO11 | 22 | Yes | - | - |
| TO12 | 24 | - | - | - |
| TO13 | 36 | Yes | - | - |
| TO14 | 22 | - | - | Yes |
| TO15 | 18 | - | Yes | Yes |
| TO16 | 9 | - | - | - |
| TO17 | 3 | - | - | - |
| TO19 | 3 | - | - | - |
| TO20 | 3 | - | - | - |
| TO21 | 7 | - | - | - |
| TO22 | 4 | - | - | - |
| TO35 | 31 | - | - | - |
| TO36 | 9 | - | - | Yes |
| MEAN 01 | 15 | - | - | - |
| MEAN 02 | 18 | Yes | Yes | - |
| MEAN 03 | 22 | - | - | - |
| MEAN 04 | 22 | - | - | - |
| MEAN 05 | 14 | - | - | - |
| MEAN 06 | 18 | Yes | - | - |
| MEAN 07 | 17 | - | - | - |
| MEAN 08 | 21 | - | - | - |
| MEAN 09 | 12 | - | - | - |
| MEAN 10 | 19 | - | - | - |
| MEAN 11 | 14 | - | - | - |
| MEAN 12 | 13 | - | - | - |
| MEAN 13 | 13 | Yes | Yes | - |
| MEAN 14 | 9 | - | - | - |
| MEAN 15 | 31 | - | - | - |
| MEAN 16 | 16 | - | - | - |
| MEAN 17 | 20 | - | - | - |
| MEAN 18 | 21 | - | - | - |
| MEAN 19 | 26 | - | - | - |
| MEAN 20 | 10 | - | - | - |
| MEAN 21 | 22 | - | - | - |
| MEAN 22 | 16 | - | - | - |
| MEAN 24 | 26 | Yes | - | - |
